# Ovarian steroid cell tumor, not otherwise specified: A rare case of postmenopausal vaginal bleeding

**DOI:** 10.3892/ol.2014.2251

**Published:** 2014-06-13

**Authors:** JING WAN, XIAOWEI CHEN, XIAOMAO LI

**Affiliations:** Department of Gynecology, The Third Affiliated Hospital of Sun Yat-sen University, Guangzhou, Guangdong 510630, P.R. China

**Keywords:** steroid cell tumor not otherwise specified, estrogen, testosterone

## Abstract

Ovarian steroid cell tumors (SCTs), not otherwise specified (NOS) are particularly rare ovarian tumors, which are composed of steroid-hormone secreting cells. The majority of patients with this tumor produce excessive quantities of testosterone and virilization is common. The current report presents a rare case of SCT in a 59-year-old female who presented with postmenopausal vaginal bleeding. The patient had experienced irregular vaginal bleeding for two months, 12 years after menopause. Transvaginal ultrasound and magnetic resonance imaging identified a solid adnexal mass and the pathological result of diagnostic curettage showed a proliferative endometrium. The patient’s serum estrogen and testosterone levels were elevated (393.71 nmol/l and 22.28 nmol/l, respectively). The patient underwent an exploratory laparotomy, hysterectomy and bisalpingectomy. The neoplasm was well-circumscribed, solid, homogeneous and yellow in color. Microscopically, the tumor was predominantly composed of granular eosinophilic or vacuolated cytoplasm. Reinke’s crystals, prominent nucleoli and Call-Exner bodies were not observed, and there was no mitotic figure. Immunohistochemistry demonstrated that the tumor cells were strongly positive for inhibin. The present rare case aims to expand the current knowledge of this type of ovarian tumor.

## Introduction

Steroid cell tumors (SCTs) of the ovary account for <0.1% of all ovarian tumors and these tumors may present at any age along with notable presentations as a result of the hormonal activity and virilizing properties of the tumor (1). To the best of our knowledge, a small number of cases of SCTs, not otherwise specified (NOS), have been described (2–11). In the current report, a case of SCT, NOS in a 59-year-old female with postmenopausal vaginal bleeding is presented. The histopathological and clinical features of the SCT are summarized and a review of the literature regarding this type of tumor is presented. The study was approved by the ethics committee of the Third Affliated Hospital of Sun Yet Sen University (Guangzhou, China). The patient provided written informed consent.

## Case report

In August 2012, a 59-year-old female (gravida 3, para 2) presented to the Department of Gynecology at The Third Affiliated Hospital of Sun Yat-sen University following two months of irregular vaginal bleeding, 12 years after menopause. A transvaginal ultrasound scan identified an enlarged uterus with a 5-mm endometrium, a 22 × 19 mm left ovarian adnexal solid mass and a small quantity of free fluid in the pelvis. The patient had undergone diagnostic curettage at the Zhaoqing First People’s Hospital (Zhaoqing, China) two weeks prior to attending The Third Affiliated Hospital of Sun Yat-sen University and the pathological result had shown a proliferative endometrium. The patient had a history of hepatitis B and diabetes mellitus, however, the systemic examination was unremarkable. A vaginal examination revealed a small amount of blood in the vagina and a small uterus, while the assessment of the adnexa of uterus was limited due to atrophy of the ovaries following menopause. A transvaginal Doppler ultrasound scan showed that the uterus was normal and the endometrium was 2-mm thick. In addition, a 30×17-mm left ovarian adnexal solid mass was observed as well as 20 mm free fluid in the pelvis. A pelvic magnetic resonance imaging (MRI) scan showed a 20×15-mm left adnexal cystic-solid mass. The patient’s liver enzyme levels were marginally elevated, with alanine aminotransferase levels of 50 U/l and a fasted blood glucose level of 7.27 mol/l. The level of cancer antigen (CA)-125 was 95.6 U/l (normal range, 0–35 U/l) and other tumor markers, including CA19-9, CA15-3, carcinoembryonic antigen and α-fetoprotein (AFP) were within the normal limits. The patient’s total serum testosterone level and estradiol (E2) level were 22.28 nmol/l (normal range, 0.5–2.6 nmol/l) and 393.71 nmol/l (normal value, <118.2 nmol/l for postmenopausal females), respectively. Normal levels of luteinizing hormone (LH), follicle-stimulating hormone (FSH) and dehydroepiandrosterone (DHEA) were observed.

**Figure 1:**
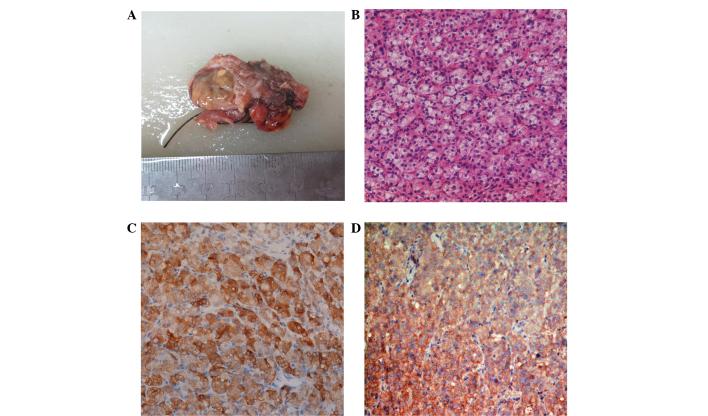
(A) Cross-section of the left ovary demonstrating the solid yellow tumor. (B) Microscopically, the tumor was composed of granular eosinophilic or vacuolated cytoplasm, which identified it as a steroid cell tumor (stain, hematoxylin and eosin; magnification, ×100). (C and D) Immunohistochemical results obtained from the ovarian tumor; magnification, ×200. The steroid cells were positive for (C) inhibin and (D) cluster of differentiation 99.

A laparotomy was undertaken and 100 ml fluid was observed in the peritoneal cavity, in addition, a solid mass measuring 3×2 cm was identified in the left ovary. The uterus, the right ovary and the fallopian tubes appeared normal. A hysterectomy and bilateral salpingo-oophorectomy were performed. The final pathology result showed a well-circumscribed tumoral mass, size 3×3 cm. The cut surface of the solid neoplasm was homogeneous and yellow. Microscopically, the tumor was predominantly composed of granular eosinophilic or vacuolated cytoplasm. Reinke’s crystals, prominent nucleoli and Call-Exner bodies were not observed, and no mitotic figure was present. Immunohistochemistry of the neoplastic cells exhibited positive staining for inhibin, however, was negative for cytokeratin (CK; Fig. 1). There was weak staining for epithelial membrane antigen (EMA), however, S-100, smooth muscle actin, CD68 and desmin were negative; thus, a final diagnosis of SCT was established. By the fourth postoperative day, the total testosterone and E2 levels fell to 3.85 nmol/l and 282.26 nmol/l, respectively. After one month, repeat assessments for total testosterone and E2 levels were conducted in the clinic and revealed normal values. The patient is currently being followed up at regular intervals.

## Discussion

SCTs of the ovary are particularly rare and account for <0.1% of all ovarian tumors (1). These tumors produce an excess of steroid hormone, specifically testosterone, therefore they are grouped into the category of sex cord-stromal tumors of the ovary. They are divided into three further subtypes according to their origins: Stromal luteoma, Leydig cell tumor and SCT, NOS, with the latter being the most common of the three subtypes, accounting for ~60% of cases (2).

Ovarian SCTs, NOS, may occur at any age, however, generally occur at younger ages compared with other SCTs and occasionally prior to puberty (9). The typical presentation, which occurs in premenopausal females (mean age, 43 years), is virilization (2); >50% of cases are clinically associated with androgenic changes. Ovarian SCT, NOS patients exhibit symptoms, including hirsutism, hair loss, amenorrhea or oligomenorrhea and ~25% of SCTs, NOS, do not produce any hormones (3). It is uncommon for SCTs, NOS, to produce hormones other than testosterone. However, the excess production of estrogen, prolactin and prorenin has previously been reported (8–10). The excess production of estrogen may result in menorrhagia and postmenopausal bleeding; in particularly rare cases, endometrial adenocarcinoma has also been documented (3,11). Hyperandrogenism accompanied by hyperestrogenism is uncommon in postmenopausal females. The testosterone level of the patient in the present study was markedly elevated and the E2 level was marginally elevated and, although the patient exhibited an estrogenic manifestation, which presented as postmenopausal bleeding and endometrial proliferation, the patient did not demonstrate the associated androgenic manifestations. However, it is possible that the changes were subtle and, thus, not clinically evident.

Elevation of testosterone levels to >200 ng/dl is the significant diagnostic threshold level for the discrimination of androgen-secreting tumors and non-neoplastic lesions. Elevated testosterone levels may result in adrenal, ovarian and iatrogenic causes and therefore, must be determined in order to diagnose or rule out disease/tumor. Elevated testosterone levels that present with normal DHEA, LH, FSH and 17-hydroxyprogesterone levels warrant the diagnosis of a virilizing ovarian tumor. MRI, endovaginal ultrasound and color flow Doppler imaging also contribute to differential diagnosis. Various other serum tumor markers (including AFP and CA-125) facilitate the differential diagnosis of ovarian adenocarcinoma (5).

The final diagnosis is usually made via the histological examination of a surgically resected specimen. The characteristic histological appearance includes the presence of diffusely arranged cells with abundant granular eosinophilic or vacuolated cytoplasm, which is often positive for fat stains, with an absence of Reinke’s crystals and stromal hyperthecosis (3,6). In addition to these microscopic features, immunohistochemistry is particularly useful for the accurate diagnosis of an SCT. Numerous immunohistochemical markers have been identified for the differential diagnosis of sex cord-stromal tumors, and inhibin was found to be particularly useful in differentiating sex cord-stromal from non-sex cord-stromal tumors (12,13). Inhibin, a hormonal polypeptide, is present in ovarian granulosa and lutein cells, as well as testicular (Sertoli’s) and Leydig cells, and suppresses the production of pituitary gonadotropins, particularly FSH. The majority of SCTs are positive for inhibin. CD99, the MIC2 gene product best known for its presence in Ewing’s sarcoma and primitive neuroectodermal tumors, is another marker that is expressed by sex cord-stromal tumors; furthermore, the CD99 antibody reacts with normal granulosa and Sertoli’s cells. Sex cord-stromal tumors are predominantly negative for CK and the majority of sex cord-stromal tumors are negative for EMA, although a small subset of tumors may be positive for EMA. Focal EMA positivity was reported in a previous case of SCT (14). In the present study, a diagnosis of sex cord SCT was made on the basis of microscopic images as well as the observation of positive immune reactivity to inhibin. Negative staining for CK7 supported a diagnosis of sex cord-stromal tumor and focal EMA positivity, which was observed in the present case, was consistent with other reported cases (14).

The majority of SCTs are benign and unilateral. In a series of 63 cases from the Massachusetts General Hospital (Boston, MA, USA), 94% of the tumors were found to be unilateral (3). However, malignancy has been reported in as high as 28.6–43% of cases and pathologic evaluation is considered to be essential for the diagnosis of a malignancy. Hayes and Scully (3) identified five pathological features of tumors that are highly associated with malignancy: i) More than two mitoses per 10 high-power field; ii) presence of necrosis; iii) diameter, ≥7 cm; iv) hemorrhaging; and v) grade 2 or 3 nuclear atypia. For the patient in the current study, the pathology result did not demonstrate any obvious signs of malignancy.

The management of SCTs is surgical removal of the tumor, with an excision of the primary lesion by unilateral oophorectomy, and is generally considered to be adequate for the treatment of young patients. As the frequency of bilateral occurrence is only 6%, and when future fertility is not an issue, hysterectomy (removal of the contralateral ovary) and complete surgical staging are recommended. However, subsequent monitoring of hormone levels is required as part of the patient’s postoperative follow-up. To the best of our knowledge, there are no reports concerning the efficacy of radiation or chemotherapy. In recent years attempts have been made to describe the use of gonadotropin-releasing hormone analogues to induce the suppression of secretions and apoptosis, which may lead to a non-surgical cure, however, these approaches have primarily been attempted with inoperable cases or patients with recurrent disease.

In conclusion, ovarian SCTs, NOS (grouped under sex chord-stromal tumors) are a particularly rare type of ovarian tumor. They are usually benign, unilateral and are characterized by hyperandrogenism and virilization. However, in a rare case, such as the present patient, hyperandrogenism and hyperestrogenism were present and the clinical manifestation was not considered to be typical. The primary treatment method was surgical extirpation of the primary lesion. We recommend that any patient who presents with high testosterone levels should be investigated systematically in order to determine whether the origin is adrenal or ovarian. An awareness of this entity will extend the appreciation of NOS ovarian steroid cell tumors.
